# Potential utility of the Tumguide® LED Light Source for safe and accurate gastric tube placement during laparoscopic gastrectomy

**DOI:** 10.1186/s40981-025-00824-6

**Published:** 2025-10-24

**Authors:** Yuto Rai, Suguru Hayase, Chiaki Nemoto, Satoki Inoue

**Affiliations:** 1 Clinical Training Center, Ohara General Hospital, 6-1 Ohomachi, Fukushima, 960-8611 Japan; 2 Department of Surgery, Ohara General Hospital, 6-1 Ohomachi, Fukushima, 960-8611 Japan; 3 Department of Anesthesiology, Fukushima Medical University, 1 Hikarigaoka, Fukushima, 960-1295 Japan; 4 Department of Anesthesiology, Ohara General Hospital, 6-1 Ohomachi, Fukushima, 960-8611 Japan

To the Editor,

Malposition of a nasogastric tube (NGT) can occasionally occur [1]. This may potentially lead to serious adverse events [2]. The Tumguide® LED Light Source (Neuroceuticals Inc., Tokyo, Japan; distributed by Otsuka Pharmaceutical Factory, Inc., Tokushima, Japan) emits highly bio-permeable red light (wavelength 660 nm) and may help identify the position of the NGT tip by allowing visual confirmation in the epigastric area from outside the body [3].

In cases of gastrectomy, the inserted NGT should be sufficiently withdrawn during resection to prevent interference with the surgical field or accidental transection. After the anastomosis is completed, the NGT should be carefully re-advanced to the appropriate position. Caution is essential to avoid blind or forceful advancement because this may injure the anastomotic site. There have been reports of adverse events in which NGTs were accidentally cut or sutured during surgery due to improper positioning [4–6].

In laparoscopic surgery, the anesthesiologist and surgeon estimate the NGT’s position based on the insertion length or by subtle movements of the gastric wall in response to tube advancement. However, these usual approaches may not always provide a reliable confirmation of the NGT’s position. Because the tube lies entirely within the stomach and is not directly visible, its position must often be inferred, and unintentional advancement can occur. We are sometimes unable to confirm the tube’s movement, and it may be unintentionally advanced further.

This case involved an 88-year-old man (height 169 cm, weight 62 kg) who underwent laparoscopic total gastrectomy for gastric cancer. In this case, the Tumguide® allowed us to easily confirm the position of the NGT tip and safely advance it to the appropriate location after anastomosis (Fig. 1). Once the Tumguide® light is turned on, it can be switched on and off as needed. However, if the total time it remains off exceeds 15 min after first activation, it will stop functioning and cannot be used again. Therefore, if we use the light to check the tube tip during stomach transection, it will no longer be available for confirming the tube’s position after anastomosis. One limitation of the Tumguide® is that it is intended for temporary, single-use only and should not be reinserted into the nasogastric tube after removal.

**Fig. 1:**
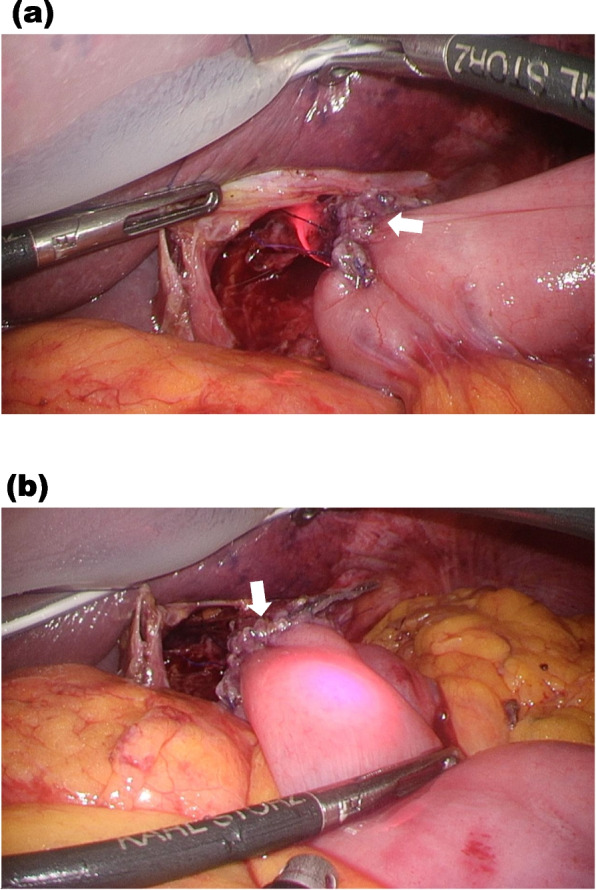
Laparoscopic view of the esophagojejunostomy site. The arrow (⇧) in each panel indicates the esophagojejunostomy site. **a** As the nasogastric tube passed through the diaphragm, the Tumguide® light became visible within the esophagus. **b** The gastric tube was advanced beyond the esophagojejunostomy into the jejunum. The red light was clearly visible through the laparoscopic camera, allowing safe confirmation of the tube’s position just distal to the anastomosis

Tumguide® allows both the surgeon and the anesthesiologist to confirm the position of the NGT intraoperatively, enabling shared recognition of its correct positioning and preventing over-insertion or excessive withdrawal.

Since the Tumguide® is a disposable and relatively expensive device, its routine use in all laparoscopic surgeries requiring NGT confirmation is impractical. From a safety management perspective, it may still be reasonable to consider the Tumguide® as an option for patients in whom NGT placement is particularly difficult or whose tube position is hard to confirm.

In this single case, we were able to successfully identify the Tumguide® during the procedure. However, care must be taken as the laparoscopic light source is very intense, and there is a risk that the Tumguide® may be overlooked.

## Data Availability

Not applicable.

## Abbreviations

NGT: Nasogastric tube
